# *Naegleria* species population found in pond water of parks in Mashhad city, Can the physicochemical factors affect it?

**DOI:** 10.1016/j.mex.2018.10.014

**Published:** 2018-10-24

**Authors:** Ali Asghar Najafpoor, Hossein Zarrinfar, Shiva Ghaderifar, Hossein Alidadi, Habibolah Esmaily, Elham Hajialilo, Bibi Razieh Hosseini Farash, Elahe Ahmadi

**Affiliations:** a Social Determinatns of Health Research Center, Mashhad University of Medical Sciences, Mashhad, Iran; b Allergy Research Center, Mashhad University of Medical Sciences, Mashhad, Iran; c Department of Environmental Health, Faculty of Health, Mashhad University of Medical Sciences, Mashhad, Iran; d Department of Biostatistics and Epidemiology, Research Health Center, Faculty of Health, Mashhad University of Medical Sciences, Mashhad, Iran; e Department of Medical Parasitology and Mycology, Faculty of Medicine, Qazvin University of Medical Sciences, Qazvin, Iran; f Department of Medical Parasitology and Mycology, Faculty of Medicine, Mashhad University of Medical Sciences, Mashhad, Iran

**Keywords:** *Naegleria* species population found in pond water of parks in Mashhad city, Can the physicochemical factors affect it?, Naegleria, Pond water, Physicochemical factors

## Abstract

*Naegleria* species are the ubiquitous free-living amoebas that are found worldwide in soil and water. Among *Naegleria* spp., *N. fowleri* can cause primary amebic meningoencephalitis (PAM). Ninety water samples were collected from the pond of parks. Also, the water quality parameters were measured at the sampling site (such as temperature, pH, total dissolved solids (TDS), electrical conductivity (EC) and Turbidity).

After filtering, the samples were cultured on Bacto-agar enriched with *Escherichia coli*. A PCR assay was conducted on the culture-positive samples in the ITS1, 5.8SrDNA and ITS2 regions, and then the PCR products were sequenced.

The pond water of parks was contaminated with some *Naegleria* spp. (except *N. fowleri*) and a *Vahlkampfia avara*. There was no significant relationship between water quality parameters and the presence of *Naegleria* (p > 0.05).

Our protocol investigates to detect *Naegleria* spp. from ponds water of parks in Mashhad city and the relations between the water quality parameters and its presence.

## Graphical abstract

**Figure:**
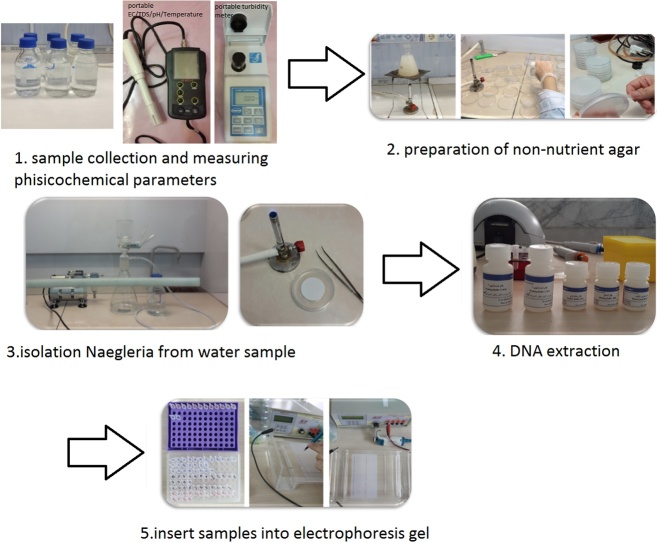

Specifications Table

**Table:** 

Subject Area	*Chemical Engineering*
More specific subject area:	Water physicochemical parameters
Protocol name:	*Naegleria* species population found in pond water of parks in Mashhad city, Can the physicochemical factors affect it?
Reagents/tools:	• Portable pH/EC/TDS/Temperature meter HANNA HI9813-5 model • Portable turbidity meter HACH 2100p model • Bacto agar powder (Canada Quelab Company) • Dyna Bio (Blood/Tissue DNA Extraction Mini Kit) IRAN Takapozist company • Thermal Cycler device gene up model
*Experimental design:	Ninety water samples were collected from the pond of parks. Also, the water quality parameters were measured *in situ* (such as temperature, pH, total dissolved solids (TDS), electrical conductivity (EC) and Turbidity). After filtering, the samples were cultured on Bacto-agar enriched with *Escherichia coli*. A PCR assay was conducted on the culture-positive samples in the ITS1, 5.8SrDNA and ITS2 regions, and then the PCR products were sequenced. The statistical analysis was performed by SPSS 16.0 software. And Fisher's Exact and Mann-Whitney test.
Ethics:	This work was as a master thesis of Shiva Ghaderifar in MUMS and was financially supported by the Deputy of Research, MUMS, Mashhad, Iran with number grant of 940310.
*Value of the Protocol:	• Mashhad is the second largest city in Iran and the cultural capital of the Islamic world and annually receives a large number of pilgrims from around the world. Ponds water of parks can be as a source of amoebic contamination for the citizens and pilgrims by routine ritual ablution and also by children who play in ponds, which can enter contaminated water into the nostrils and causes PAM [1]. It is worthy to mention most of PAM cases have been reported in healthy people with a history of water contacts [2]. Water supplies can be potential sources of Naegleria and individuals probably encounter this parasite during our daily life involved with the water [3]. • Therefore, identifying the locations containing *Naegleria* is important to take preventive measures. This requires accurate and reliable methods such as molecular techniques to find and detect [4]. Identification of *Naegleria* is mainly based on cultivation on non-nutrient agar enriched with heat-killed *Escherichia coli*, and molecular methods [1]. The accuracy of culture along with direct microscopy is less than PCR method to identify of amoeba [2]. PCR is more sensitive technique than direct microscopy of culture, but the use of both PCR and culture method is suggested for environmental water samples to gain more complete results of the real presence of *Naegleria* [2]. Hence, the current study used culture and PCR method for detection of *Naegleria* spp. from ponds water of parks; also, investigate the relations between the water quality parameters and the presence or absence of *Naegleria*.

## Description of protocol

### Sample collection

Sampling was done from ponds water of parks in Mashhad city during August to December 2015. A total of 90 water samples were collected. The samples were taken from different park's ponds of 13 regions of Mashhad. To take water samples and sampling requirements standard method book, was utilized [3]. Physicochemical parameters such as temperature, pH, total dissolved solids (TDS) and electrical conductivity (EC) were measured at the sampling sites by portable pH/EC/TDS/Temperature meter HANNA HI9813-5 model and Turbidity by portable turbidity meter HACH 2100p model.

### Preparation of non-nutrient agar

To preparation 1.5% Non-nutrient agar (NNA), 1.5 g of Bacto agar powder (Canada Quelab Company) were dissolved in 99 ml distilled water, and 1 ml ameba page saline was added (Table 1). The solution was autoclaved for 15 min at a temperature of 121 °C. Then it was spread on plates and was kept in the refrigerator (4 °C) until using them.

**Table 1:** Preparation of ameba Page saline.

Amount	Material
120 mg	NaCl
4 mg	MgSo4,7H2O
4 mg	CaCl2, 2H2O
142 mg	Na2HPO4
146 mg	KH2PO4
1 L	Distilled water

### Isolation *Naegleria* from water samples

Approximately 500 ml of water samples were filtered through nitrocellulose membranes with 0.45 μm pore-size. Then, the filters were cultured on 1.5% NNA medium which was enriched with *Escherichia coli*. The plates were incubated at room temperature, after at least four days each plate was examined daily under a light microscope to check the presence of free-living *amoebae* (FLA) [4].

The plates were kept one month, and they were considered negative in the absence of amoebas after this period. All of the plates were examined with a stereomicroscope for the trophozoite and cystic forms as positive plates. A piece of NNA medium agar which contained the amoebas in positive plates was chosen and placed on the solidified fresh NNA medium. The plates were incubated at 30 °C and were monitored daily for *Naegleria* growth. Distilled water (about 2000 microlitrs) were added to positive samples. Then, the surface and beneath of agar was scratched with lam edge to isolate *Naegleria*. Isolated *Naegleria* were collected in 1.5 ml micro centrifuge tubes. They were centrifuged for 10 min at 10,000 rpm to remove additional agar.

### DNA extraction and PCR

Commercial Dyna Bio Genomic Mini Kit (Blood/Tissue DNA Extraction Mini Kit) was used to extract DNA according to the manufacturer’s protocol. Then, the DNA was stored at −20 °C.

The PCR reaction was performed using the primers as described by Panda et al. in the internal transcribed spacers (ITS) 1, 5.8SrDNA and ITS2 regions (Table 2) [5].

**Table 2:** Primers sequence to amplify of the internal transcribed spacers (ITS) 1, 5.8SrDNA and ITS2 for *Naegleria* genus and *Naegleria fowleri* species.

Reverse primer (3′ → 5′)	Forward primer (3′ → 5′)	Free-living amoebae (FLA)
AAATAAAAGATTGACCATTTGAAA	GTGAAAACCTTTTTTCCATTTACA	*Naegleria fowleri*
TTTCTTTTCCTCCCCTTATTA	GAACCTGCGTAGGGATCATTT	*Naegleria* genus

The *expected* size for *amplification* (*PCR product*) of *Naegleria* genus was about 400–450 bp and for *N*. *fowleri* it was about 300 bp. The amplification reaction mixture consisted of 10 μl of Taq DNA Polymerase *Master Mix* red (2×; Ampliqon, Denmark), 7 μl distilled water, 1.5 μl of each primer (5 mM) and 1.5 μl DNA in a total volume of 20 μl were placed in a Thermal Cycler device. Every PCR run contained a tube with distilled water instead of template DNA as a negative control. The cycles of PCR were set up as follows: first denaturation step at 95 °C for 5 min and 35 cycles of denaturation at 95 °C for 30 s, annealing at 54 °C for 30 s and extension at 72 °C for 1 min and a final extension step at 72 °C for 5 min. The PCR-products electrophoresis was done on 2% agarose gel, stained with the green viewer and visualized under blue light. The PCR products were then sequenced to confirm the amplified fragment and genotypic identification [6].

The results showed the presence of various species of *Naegleria* in ponds water of parks in Mashhad city. Eighteen (20%) of the 90 samples were positive for *Naegleria* spp. including *N. Americana, N. clarki, N. andersoni, N. fultoni, N. carteri* and *N. pagei.* And also, one *Vahlkampfia avara* was isolated. But *N. fowleri* was not detected in any samples. Although these species are non-pathogenic, they can serve as vehicles for facultative pathogens and can be a potential public health threat. According to the statistical analysis of the results, there was no significant relationship between water quality parameters and the presence of *Naegleria* (p > 0.05).

## References

[1] Visvesvara G.S., Moura H., Schuster F.L. (2007). Pathogenic and opportunistic free-living amoebae: Acanthamoeba spp., Balamuthia mandrillaris, Naegleria fowleri, and Sappinia diploidea. FEMS Immunol. Med. Microbiol..

[2] Magnet A., Fenoy S., Galván A., Izquierdo F., Rueda C., Vadillo C.F. (2013). A year long study of the presence of free living amoeba in Spain. Water Res..

[3] Water Environment Federation, American Public Health Association (2005). Standard Methods for the Examination of Water and Wastewater.

[4] Di Filippo M.M., Santoro M., Lovreglio P., Monno R., Capolongo C., Calia C. (2015). Isolation and molecular characterization of free-living Amoebae from different water sources in Italy. Int. J. Environ. Res. Public Health.

[5] Panda A., Khalil S., Mirdha B.R., Singh Y., Kaushik S. (2015). Prevalence of Naegleria fowleri in environmental samples from northern part of India. PLoS One.

[6] Ghaderifar S., Najafpoor A.A., Zarrinfar H., Esmaily E., Hajialilo E. (2018). Isolation and identification of Acanthamoeba from pond water of parks in a tropical and subtropical region in the middle east, and its relation with physicochemical parameters. BMC microbiology.

